# Genotypic spectrum of *ABCA4*-associated retinal degenerations in 211 unrelated Mexican patients: identification of 22 novel disease-causing variants

**DOI:** 10.1007/s00438-024-02174-x

**Published:** 2024-08-20

**Authors:** Oscar F. Chacon-Camacho, Nancy Xilotl-de Jesús, Ernesto Calderón-Martínez, Vianey Ordoñez-Labastida, M. Isabel Neria-Gonzalez, Rocío Villafuerte-de la Cruz, Augusto Martinez-Rojas, Juan Carlos Zenteno

**Affiliations:** 1 Department of Genetics, Institute of Ophthalmology “Conde de Valenciana”, Chimalpopoca 14, Col. Obrera, Cuauhtemoc, Mexico City, CP06800 Mexico; 2 Laboratorio 5, Edificio A-4, Carrera de Médico Cirujano, Facultad de Estudios Superiores Iztacala, Universidad Nacional Autónoma de México, Laboratorio 5, Edificio A-4, Mexico City, Mexico; 3 Department of Biomedical Informatics, Faculty of Medicine, National Autonomous University of Mexico (UNAM), Mexico City, Mexico; 4 Rare Disease Diagnostic Unit, Faculty of Medicine, National Autonomous University of Mexico (UNAM), Mexico City, Mexico; 5 Faculty of Medicine, Autonomous University of the State of Morelos (UAEM), Morelos, Mexico; 6 Laboratory of Integrative Microbiology and Molecular Biology, Division of Chemical and Biochemical Engineering, TecNm: Tecnológico de Estudios Superiores de Ecatepec. Ecatepec de Morelos, Morelos, Edo. México Mexico; 7 Tecnologico de Monterrey, Escuela de Medicina y Ciencias de la Salud, Monterrey, Mexico; 8 Biochemistry Department, Faculty of Medicine, UNAM, Mexico City, Mexico

**Keywords:** Retinal dystrophy, Stargardt’s disease, *ABCA4*, Founder mutation

## Abstract

The purpose of this study was to analyze and molecularly describe the largest group of patients with ABCA4-associated retinal degeneration in Latin America. Pathogenic variants in *ABCA4*, a member of the ATP Binding Cassette (ABC) transporters superfamily, is one of the most common causes of inherited visual deficiency in humans. Retinal phenotypes associated with genetic defects in *ABCA4* are collectively known as ABCA4-associated retinal degenerations (ABCA4R), a group of recessively inherited disorders associated with a high allelic heterogeneity. While large groups of Caucasian and Asiatic individuals suffering from ABCA4R have been well characterized, molecular information from certain ethnic groups is limited or unavailable, precluding a more realistic knowledge of ABCA4-related mutational profile worldwide. In this study, we describe the molecular findings of a large group of 211 ABCA4R index cases from Mexico. Genotyping was performed using either next generation sequencing (NGS) of a retinal dystrophy genes panel or exome. *ABCA4* targeted mutation testing was applied to a subgroup of subjects in whom founder mutations were suspected. A total of 128 different *ABCA4* pathogenic variants were identified, including 22 previously unpublished variants. The most common type of genetic variation was single nucleotide substitutions which occurred in 92.7% (408/440 alleles). According to the predicted protein effect, the most frequent variant type was missense, occurring in 83.5% of disease-causing alleles (368/440). Mutations such as p.Ala1773Val are fully demonstrated as a founder effect in native inhabitants of certain regions of Mexico. This study also gives us certain indications of other founder effects that need to be further studied in the near future. This is the largest molecularly characterized ABCA4R Latin American cohort, and our results supports the value of conducting genetic screening in underrepresented populations for a better knowledge of the mutational profile leading to monogenic diseases.

**Supplementary Information:**

The online version contains supplementary material available at 10.1007/s00438-024-02174-x.

## Introduction

Molecular-genetic studies, including next-generation sequencing, have recently made it possible to characterize populations for monogenic diseases with genetic heterogeneity or for huge genes, such as *ABCA4*. These studies related to ABCA4R are scarce and with few cases described in Mexico, although recently Zenteno et al. in a genetic study of retinal dystrophies demonstrated that this gene is one of the most frequent in our population (Zenteno et al. 2020). Our cohort is the largest in Latin America and will allow us to know the most frequent pathogenic variants in the country, so that we can establish founder effects dependent on the region studied in Mexico, thus demonstrating the great genetic diversity of our population. ATP Binding Cassette (ABC) transporters are members of a protein superfamily existing in all organisms and responsible for obtaining energy from ATP binding and hydrolysis in order to drive transport of substrates across biological membranes (Thomas and Tampé 2020). The ABCA4 (subfamily A, member 4) protein is encoded by *ABCA4*, a gene located in 1p22.1 and composed by 50 coding exons. ABCA4 is a specific transporter that imports N-retinylidene phosphatidyl-ethanolamine (NrPE) and phosphatidylethanolamine (PE), between the luminal side and the cytoplasm of the outer segment of the rod and cone photoreceptor discs (Quazi et al. 2012) Genetic defects in *ABCA4* gene can lead to retinal accumulation of N-retinyl-N-retinylidene ethanolamine (A2E), a major component of lipofuscin and with toxic effects in photoreceptors and retinal pigment epithelium (RPE) cells. The toxic activity of A2E and bisretinoids cause an inflammatory effect that activates the complement system and the inflammasomes, leading to the death of photoreceptor cells (Sparrow et al. 2008). Pathogenic variants in *ABCA4* results in a group of retinal phenotypes, collectively referred as ABCA4-associated retinal degenerations (ABCA4R) which are characterized by a notable clinical and allelic heterogeneity (Molday and Zhang 2010). For most cases, ABCA4R manifests in the first or second decade of life as Stargardt disease (STGD1), a disorder distinguished by central vision loss, dyschromatopsia, and photophobia (Fishman et al. 1999; Rotenstreich et al. 2003). However, ABCA4R cand also include other phenotypes such as bull’s-eye maculopathy (Duncker et al. 2015), retinitis pigmentosa (Fukui et al. 2002), rod-cone dystrophy (Simonelli et al. 2004), choriocapilaris dystrophy (Bertelsen et al. 2014), and early-onset chorioretinopathy (Tanaka et al. 2018). Clinical diagnosis can be complicated as other inherited retinal dystrophies may have a similar phenotype as ABCA4R, termed phenocopies, with variants in the *PRPH2* gene being the most commonly recognized subtype (Boon et al. 2007; Cremers et al. 2020; Ibanez et al. 2021). To date, ∼ 1200 different pathogenic variants distributed throughout the entire *ABCA4* gene have been identified in subjects with ABCA4R worldwide (Stenson et al. 2020). While a number of studies have allowed a comprehensive characterization of the clinical and mutational features of ABCA4R patients from North America, Western Europe, and Eastern Asia (Al-khuzaei et al. 2021), there are few studies carried out in Latin American (Chacón-Camacho et al., 2013; Salles et al. 2018; Mena et al. 2021). Understudied populations may display a distinct ABCA4R allelic architecture and thus represent a possible source for the identification of both novel disease causing-variants and of specific founder mutation effect(s). In this study, we describe the molecular findings of a large group of 211 ABCA4R index cases from Mexico. The identification of 22 previously unpublished *ABCA4* pathogenic variants in this cohort supports the value of conducting genetic screening in underrepresented populations for a better knowledge of the mutational profile leading to monogenic diseases.

## Materials and methods

This study was approved by the Institutional Review Board of “Conde de Valenciana” Institute of Ophthalmology at Mexico City (IRB CI-034-2021) and adhered to the tenets of the Declaration of Helsinki. Written consent was obtained from all participants (probands and relatives) or their parents when indicated. For this study, all probands were recruited from 2013 to 2023, and were subjects originating from various regions of Mexico. From the total number of probands with mutations in the *ABCA4* gene, 110 individuals were analyzed by gene panel sequencing, 55 by exome sequencing, 24 by Sanger sequencing of the complete *ABCA4* gene, and 15 by partial *ABCA4* Sanger sequencing guided by geographic origin of patients (founder mutations). The remaining patients were genetically characterized by a combination of either gene panel sequencing/targeted analysis of founder mutations (5 cases) or Sanger sequencing/gene panel sequencing (2 cases). All probands underwent a complete ophthalmological evaluation, including best corrected visual acuity, biomicroscopy, color fundus photography, fundus autofluorescence, electrophysiological testing, and swept-source optical coherence tomography. A retina specialist gave clinical diagnosis, based on patients’ visual symptoms history, ocular clinical examination, retinal imaging, and electrophysiology findings. Information about possibly affected family members was also collected. Clinical diagnoses were carried out according to the phenotypic heterogeneity of the ABCA4R described previously: Stargardt disease, cone-rod dystrophy, retinitis pigmentosa, maculopathy (Al-Khuzaei et al., 2021). Sporadic cases were assumed in pedigrees with single affected individuals while familial cases were considered when there were two or more affected individuals in a family. To rule out syndromic entities, systemic examination was performed by a geneticist. All patients included in the study were of Mexican-mestizo descent.

### DNA extraction

Genomic DNA (gDNA) was extracted from peripheral blood leukocytes of all participants using a QIAaMP DNA Blood kit (Qiagen, Hilden, Germany). gDNA integrity was assessed through gel electrophoresis while quantification and purity of samples were measured employing Nanodrop 2000 spectrophotometer (Thermo Fisher Scientific, Waltham, MA, USA). For genotyping, either next generation sequencing (NGS) of a retinal dystrophy genes panel or exome sequencing were performed.

### Inherited retinal disorders panel (Invitae, San Francisco, CA, USA)

gDNA obtained was enriched for targeted regions using a hybridization-based protocol and the resulting libraries sequenced using Illumina technology. All targeted regions were sequenced at > 50x depth. Reads were aligned to a reference sequence (GRCh37) and sequenced changes were identified and interpreted in the context of the canonical *ABCA4* transcript. The sequencing method was able to detect insertions and deletions larger than 15 bp and copy number variations (CNVs) at a single exon resolution at virtually all targeted *ABCA4* exons. Designation of pathogenic and likely pathogenic variants was made according to the American College of Genetics and Genomics (ACMG) guidelines (Richards et al. 2015).

### Whole exome sequencing

Briefly, exon regions of all human genes (∼22,000) were captured by xGen Exome Research Panel v2 (Integrated DNA Technologies, Coralville, Iowa, USA). The captured regions of the genome were sequenced using a commercial service (3 billion Inc., Seoul, South Korea) employing a Novaseq 6000 instrument (Illumina, San Diego, CA, USA). The raw genome sequencing data analysis, including alignment to the GRCh37/hg19 human reference genome; variant calling and annotation was conducted with open-source bioinformatics tools and an in-house software. The automatic variant interpretation software EVIDENCE (Seo et al. 2020), was employed to prioritize variants based on ACMG guidelines (Richards et al. 2015). The average percentage of coverage was 98% to 100X. Exome sequencing data (VCF files) were filtered and analyzed using the Franklin Genoox tool (https://franklin.genoox.com) for variant prioritization (Jackson et al. 2024).

### Sanger sequencing

All candidate disease-causing variants identified by NGS were confirmed by Sanger sequencing; in addition, familial co-segregation analysis of disease-causing variants was carried out also by Sanger sequencing on available relatives for pathogenicity support. Briefly, PCR products (primers for amplification are available upon request) were purified and sequenced using the BrilliantDye Terminator v1.1 Cycle Sequencing kit (NimaGen BV, Netherlands), as previously described (Chacón-Camacho et al. 2013). Samples were analyzed in a Spectrum Compact CE System sequencer (Promega Corporation, WI, USA). All variants were submitted to two recognized databases ClinVar Submission portal of the National Library of Medicine (NIH) (https://submit.ncbi.nlm.nih.gov/clinvar), and LOVD (Leiden Open Variation Database) (https://databases.lovd.nl/shared/users?register).

#### Interpretation of the sequence variants

All variants identified in this study were classified following the criteria of the American College of Medical Genetics and Genomics and the Association for Molecular Pathology. According to these criteria, the variants were classified as pathogenic if: (a) they have 1 or more very strong criteria (PVS1), and 1 or more strong (PS1-PS4), or 2 or more moderate (PM1-PM6), or 1 moderate (PM1-PM6) and 1 support (PP1-PP5); (b) 2 or more strong criteria (PS1-PS4); (c) 1 strong criteria (PS1-PS4) and, 3 or more moderate (PM1-PM6), or 2 moderate (PM1-PM6) and 2 supportive (PP1-PP5), or 1 moderate (PM1-PM6) and 4 or more supportive (PP1-PP5). To consider a genetic variant as probably pathogenic, it needs to meet: (a) 1 very strong criteria (PVS1) and 1 moderate (PM1-PM6), (b) 1 strong criteria (PS1-PS4) and 1–2 moderate (PM1-PM6), (c) 1 strong (PS1-PS4) and 2 or more supportive (PP1-PP5), (d) 3 or more moderate (PM1-PM6), (e) 2 moderate (PM1-PM6) and 2 or more supportive (PP1-PP5 ), and (f) 1 moderate (PM1-PM6) and 4 or more supportive (PP1-PP5). For more details, you may consult the guide for the interpretation of variants (Richards et al. 2015).

## Results

A total of 211 unrelated probands were demonstrated to carry biallelic pathogenic/likely pathogenic mutations in *ABCA4* (Supplementary Table 1). A total of 101 additional individuals were sequenced in this study, 86 of them were relatives of positive ABCA4Rs (29 affected relatives, 51 carriers and 6 healthy), while 15 of them were negative for ABCA4, but all of them had mutations in other genes related to hereditary retinal dystrophies. Therefore, a total of 330 patients were sequenced. Of 211 solved cases, a total of 98 probands (46.5%) were males, while 53.5% were females; only 39 probands (18.5%) pertained to pedigrees with two or more affected individuals while 172 (81.5%) were sporadic. 90% (190/211) of molecularly solved patients had a clinical diagnosis of STGD1, 4% (9/211) had a clinical diagnosis of cone dystrophy, 3% (6/211) had retinitis pigmentosa, and 3% (6/211) had an unspecified macular dystrophy.While a total of 211 index cases had biallelic pathogenic variants in *ABCA4* confirming a ABCA4R diagnosis, a subgroup of 18 patients were shown to carry a single disease-causing variant in this gene and thus were considered as unsolved (data not shown). Thus, a total diagnostic rate of 86.47% (211 *ABCA4* positive probands / 18 *ABCA4* heterozygous probandss / 16 *ABCA4* negative probands) was obtained. An additional group of 86 relatives from ABCA4R index cases were molecularly analyzed allowing the identification of 29 subjects with biallelic *ABCA4* mutations, 51 healthy carriers, and 6 wild-type subjects.

### Spectrum of ABCA4 pathogenic variants

A total of 128 different *ABCA4* pathogenic variants were identified (Suppl. Table 2). Including 17 complex alleles, a total of 440 disease-associated variants were characterized in the cohort of 211 probands. The most frequently affected *ABCA4* exons were exon 38 (96/440 pathogenic alleles; 22%), exon 35 (47/440; 11%), exon 16 (37/440; 8.5%), exon 23 (23/440; 5%), and exon 30 (19/440; 4%). No pathogenic variants were recognized in exons 10, 25, 37, 40, 49, and 50. A total of 440 disease-associated variants in 422 alleles were identified in this group of ABCA4R patients, including 17 instances of complex alleles (suppl. Table 3). Single nucleotide substitutions were the most common type of genetic variation occurring in 408/440 alleles (92.5%), followed by 30 small duplications/deletions (7%), and two gross intragenic deletions (0.5%). According to the predicted protein effect, the most frequent types of variants were missense (368/440 variants; alleles; 83.5%), splice-altering (24/440; 5.5%), frameshift (21/440; 5%), stop gain (17/440; 4%), and other variants (10/440; 2%).*ABCA4* genotype in 76% of patients (160/211) was compound heterozygous while 24% (51/211) was homozygous. Of note, a homozygous genotype was demonstrated to arise from paternal chromosome 1 uniparental disomy (Villafuerte-De la Cruz et al. 2022). The most commonly identified pathogenic variant was c.5318 C > T, which predicts a missense p.Ala1773Val mutation and had an allelic frequency of 79/440 (18%). Other common *ABCA4* variants were c.2453G >A (p.Gly818Glu; 32/440, 7%), c.4854G >C (p.Trp1618Cys; 20/440, 4.5%), and c.3386G >T (p.Arg1129Leu; 19/440, 4%) (Table 1). Two pathogenic deep intronic variants, c.4352+61G >A (2 alleles) and c.5196+1137G >A (3 alleles), were identified, which corresponds to 1% of all disease-causing alleles. A total of 22 previously unpublished *ABCA4* disease-causing variants, including 11 single nucleotide substitutions, were recognized in this study (Table 2). Of note, except for one allele occurring twice (c.3814-2 A >T), all these novel *ABCA4* variants were observed once, indicating that they are very rare alleles in our population. Among these previously unpublished variants, a deletion of the entire exon 7 and a deletion covering exons 17 to 23 were also characterized.

**Table 1:** Most frequent variants identified in the ABCA4 gene in our study

Variant #	Nucleotide variant (cDNA)	Protein variant	Allelic frequency (%)
1	c.5318 C > T	p.Ala1773Val	79 (17.96)
2	c.2453G > A	p.Trp1618Cys	32 (7.27)
3	c.4854G > C	p.Arg1129Leu	20 (4.55)
4	c.4919G > A	p.Arg1640Gln	17 (3.86)
5	c.6221G > T	p.Gly2074Val	14 (3.18)
6	c.5324T > A	p.Ile1775Asn	13 (2.96)
7	c.634 C > T	p.Arg212Cys	12 (2.73)
8	c.1804 C > T	p.Arg602Trp	9 (2.05)
9	c.4457 C > T	p.Pro1486Leu	8 (1.82)
10	c.4667G > C	p.Arg1556Thr	7 (1.59)
11	c.5882G > A	p.Gly1961Glu	7 (1.59)

**Table 2:** Previously Unpublished ABCA4 variants identified in our study

Variant #	Allelic frequency	Nucleotide variant (cDNA)	Protein variant	Location (E: exón; I:intrón)	ACMG classification
1	1	c.438delT	p.Ile146fs	E-4	Likely pathogenic
2	1	c.689G > T	p.Cys230Phe	E-6	Likely pathogenic
3	1	c.768 + 1G > A		I-5	Likely pathogenic
4	1	c.6446G > C	p.Arg2149Pro	E-47	Pathogenic
5	1	c.1876_1888del	p.Ala626Leufs*19	E-13	Pathogenic
6	1	c.1994del	p.Tyr665fs	E-14	Pathogenic
7	1	c.2396 C > T	p.Pro799Leu	E-16	Likely pathogenic
8	1	c.2453G > C	p.Gly818Ala	E-16	Pathogenic
9	1	c.2522_2530del	p.Gln841_Met843del	E-16	Likely pathogenic
10	1	deletion		E17-23	Pathogenic
11	1	c.2741_2742del	p.His914Argfs*5	E-18	Pathogenic
12	1	c.2807del	p.Lys936Argfs*14	E-19	Pathogenic
13	1	c.2908del	p.Thr970Profs*7	E-19	Pathogenic
14	1	c.3608-1G > A		I-25	Pathogenic
15	2	c.3814–2 A > T		I-26	Pathogenic
16	1	c.3898del	p.Arg1300Aspfs*89	E-27	Pathogenic
17	1	c.4243dup	p.Thr1415Asnfs*7	E-28	Pathogenic
18	1	c.4313 C > A	p.Pro1438Gln	E-29	Likely pathogenic
19	1	c.5460 + 3G > A		I-37	Likely pathogenic
20	1	c.6401 A > G	p.Glu2134Gly	E-47	Pathogenic
21	1	Deletion		E-7	Likely pathogenic
22	1	c.6308 C > A	p.Pro2103His	E-46	Pathogenic

## Discussion

In this work, the largest cohort (211 index cases) of Latin American patients with retinal dystrophies due to *ABCA4* pathogenic variants is reported. Only 3 previous studies have been reproduced in Latin America. Chacón-Camacho et al., in 2013 reported a pilot study of patients with Stargardt disease, described a diagnostic rate of 65% and a possible founder effect for the p.Ala1773Val mutation in the central region of Mexico (Chacon-Camacho et al., 2013). In 2018, Salles et al., in a cohort of 44 Brazilian families, reported a diagnostic rate of 80% for the ABCA4 gene. In a cohort of 95 Argentine probands, Mena et al. described a diagnostic rate of 73% and two frequent mutations, p.Gly1961Glu and p.Arg1129Leu. The most frequent mutation identified in the Brazilian population was p.Arg602Trp with an allelic frequency of 12%, but is relatively rare in our population (< 2%). In the other hand, the mutations seen in the Argentine cohort were p.Gly1961Glu and Arg1129Leu, which represented 20% of the all mutations identified in this population. In addition, these pathogenic variants are frequent in populations from central Europe and Spain, respectively. In the Mexican population, the p.Gly1961Glu mutation is rare < 1.6%, while the Arg1129Leu mutation has a higher frequency of 4.1%, but the latter is frequent in the northern region of Mexico, which makes us think about possible effects of migration of the Spanish population in Mexico and Argentina. (Chacón-Camacho et al., 2013; Salles et al. 2018; Mena et al. 2021). In this new study we have a ABCA4 diagnostic rate of 86.47%, compared to the previous study where we had found 65% (Chacón-Camacho et al., 2013), it demonstrates the power of NGS technologies to increase diagnostic rates.A total of 22 previously unpublished ABCA4 pathogenic/likely pathogenic variants were characterized in this group, with half of the being single nucleotide substitutions. Interestingly, virtually all these variants occurred as single alleles, indicating that they are an uncommon cause of ABCA4R in our population.While ABCA4R are associated with extensive phenotypic variability, the most common clinical categories are STGD1, cone dystrophy, and retinitis pigmentosa (Del Pozo-Valero et al. 2020; Sheffield and Stone 2011; Tracewska et al. 2019). The variability of these phenotypes may be related to the degree of residual functional enzymatic activity associated with the genotype, to the fact that cones are more damaged than rods in A2E accumulation, and/or to the fact that RPE disturbances result in secondary damage in both cones and rods. Previously, a genotypic-phenotypic correlation was proposed in which the presence of two “severe” (loss of function) variants were most probably associated with profound photoreceptor dysfunction as occurs in RP and cone dystrophy, and one mild (missense) mutation together with a severe one or two “moderate” (missense, in frame) mutations leading to STGD1 (Klevering et al. 2005). In most of our patients with retinitis pigmentosa and cone-rod dystrophy, at least one allele carried a frameshift or a stop codon predicting the formation of a truncated protein, which could explain the severe retinal phenotype, including variants p.Asp1128, p.Ile1602Tyr*8, p.Ala1598Asp, exon 7 deletion, p.Arg 408*, and Trp1479*. Some of these mutations have been previously described in association with retinitis pigmentosa or cone-rod dystrophies, as p.Asp1128Gly (Del Pozo-Valero et al. 2020), p.Arg1640Trp (Shroyer et al. 2001; Valverde et al. 2007), p.Arg408* (Wiszniewski et al. 2005), p.1602Tyrfs*8 (Zenteno et al. 2020), p.Ala1598Asp (Boulanger-Scemama et al. 2015; Maugeri et al. 2000), and c.6282+3 A >T (Zenteno et al. 2020). Interestingly, many of the variants related to RP and cone dystrophy are variants that do not occurred in STGD1 in our population.The vast majority of ABCA4R clinical phenotypes in the present work was compatible with STGD1. Recent studies showed a similar ABCA4R-related phenotypic proportion, as observed in a cohort of 116 Chinese patients with ABCA4R which included 100 patients with STGD1 (86%), 11 with cone dystrophy (9.5%), and 4 with retinitis pigmentosa (3.5%) (Sun et al. 2021). However, in other studies the percentage of retinitis pigmentosa or cone dystrophy due to *ABCA4* mutations may be higher (Sung et al. 2020), mainly due to founder mutation effects and/or due to cohort particularities. While NGS technologies have greatly improved the molecular diagnosis in patients with inherited retinal dystrophies, it is desirable to develop cheaper strategies for genetic characterization. In our population, we have successfully applied an *ABCA4* mutation detection method depending on the place of origin of patients, by taking advantage of the knowledge of *ABCA4* founder mutations occurring in those regions. Using this fast and unexpensive method, we were able to detect causal *ABCA4* mutations by Sanger sequencing a few *ABCA4* exons in at least 20 probands from our cohort, particularly for patients originating from the central region of Mexico where the p.Ala1773Val founder variant is highly prevalent (Chacón-Camacho et al. 2013; Cremers et al. 2020; López-Rubio et al. 2018).The most frequently identified mutation in our cohort was c.5318C >T (p.Ala1773Val), observed at an allelic frequency of 18% (79/440 alleles). Thus, it can be concluded that approximately 1 in 5 Mexican ABCA4R patients carry this pathogenic variant. The p.Ala1773Val mutation has recently been described in Chinese STGD1 families (Zhu et al. 2021). It is currently unknown if the p.Ala1773Val variant has a common ancestral origin or if it arose independently in different ethnic groups and haplotype studies will be required to address this issue.Other common ABCA4 pathogenic variants in our cohort included c.2453G > A (p.Gly818Glu) with an allele frequency of 7%, c.4853G > C (p.Trp1618Cys; 4.5%), and c.3386G > T (p.Arg1129Leu; 4%). Interestingly, the p.Arg1129Leu variant is the most common ABCA4R-related variant in Spanish population where it is observed at an allelic frequency of 19% (Del Pozo-Valero et al. 2020; Valverde et al. 2007), indicating that it could represent an ancestral mutation introduced to Mexico from Spanish immigrants. The p.Gly818Glu and p.Trp1618Cys variants were also frequent in our cohort, although they have a very low frequency in other populations (Allikmets et al. 1997; Zenteno et al. 2020).Two novel intragenic deletions and two already known deep intronic variants were identified in *ABCA4* in our cohort. Particularly, a deletion encompassing exons 17–23 and an exon 7 deletion, allowed for the expansion of the CNV spectrum in ABCA4R (Al-khuzaei et al. 2021; Cremers et al. 2020). Deep intronic variants, have been identified in up to 4% of *ABCA4* alleles in different reports (Cremers et al. 2020), and in 15–40% of patients where just a single pathogenic variant was identified at first screening (Braun et al. 2013; Sangermano et al. 2019). In our cohort, only 5 deep intronic variants (1%) were identified. The c.5196 + 1137G > A mutation, identified in 3 alleles from our group, is one of the most frequent deep intronic variants and it has been demonstrated to cause functional defects by in vitro splice assays (Bauwens et al. 2019; Khan et al. 2020). The c.4352 + 61G > A mutation (identified in two alleles from our cohort) has a severe effect on RNA, although it is less frequent than the c.5196 + 1137G > A variant (Bertelsen et al. 2014; Khan et al. 2020). Complex alleles were detected in 17 patients (8%), a percentage very similar as reported in previous studies (Shroyer et al. 2001; Zhang et al. 2015). In conclusion, we describe the largest molecularly characterized ABCA4R cohort to date in Latin America and our results confirm p.Ala1773Val as a *ABCA4* founder mutation with a high prevalence in Mexican STGD1 individuals. We identified other possible autochthonous founder effects and also founder effects of other Mexican mestizo ethnic groups of European origin, which is very interesting and will be the objective of a future study to be carried out by our research group. We expanded the spectrum of *ABCA4*-disease causing variants by the recognition of 22 previously unpublished disease-causing variants, supporting the value of genetic screening in underrepresented populations for a better knowledge of the mutational profile leading to monogenic diseases.

### Electronic supplementary material

Below is the link to the electronic supplementary material.

Supplementary Material 1

Supplementary Material 2

Supplementary Material 3

Supplementary Material 4

## Data Availability

The raw NGS datasets generated during the current study are not openly available due to ethical concerns but are available from the corresponding author upon reasonable request.
