# Supplementary material for: Genotypic spectrum of ABCA4-associated retinal degenerations in 211 unrelated Mexican patients: identification of 22 novel disease-causing variants
Source: Mol Genet Genomics. 2024 Aug 20;299(1):79. doi: 10.1007/s00438-024-02174-x (PMC11335775; doi:10.1007/s00438-024-02174-x)
Supplement: Supplementary file 1 — Supplementary Material 1 [file 438_2024_2174_MOESM1_ESM.docx]

**Supplementary table 1.** *ABCA4* genotypes in 211 Mexican patients

| **Patient #** | **ID** | **Allele 1** | **Allele 2** | **Zygosity** | **Age**  **(years)** | **Phenotype** |
| --- | --- | --- | --- | --- | --- | --- |
| 1 | 269 | c.5318C>T,p.Ala1773Val | c.5318C>T,p.Ala1773Val | homozygous | 25 | Stargardt Disease |
| 2 | 279 | c.868C>T,p.Arg290Trp | c.768+1G>A | compound heterozygous | 35 | Stargardt Disease |
| 3 | 376 | c.634C>T,p.Arg212Cys | c.5318C>T,p.Ala1773Val | compound heterozygous | 22 | Stargardt Disease |
| 4 | 720 | c.3386G>T,p.Arg1129Leu | c.4880del,p.Leu1627Argfs*35 | compound heterozygous | 19 | Stargardt Disease |
| 5 | 769 | c.5044_5058del,p.Val1682_Val1686del | c.4328G>A,p.Arg1443His | compound heterozygous | 16 | Stargardt Disease |
| 6 | 793 | c.4249_4251del,p.Phe1417del | c.5882G>A,p.Gly1961Glu | compound heterozygous | 62 | Stargardt Disease |
| 7 | 931 | c.5318C>T,p.Ala1773Val | c.5318C>T,p.Ala1773Val | homozygous | 30 | Stargardt Disease |
| 8 | 1014 | c.3041T>G,p.Leu1014Arg | c.52C>T,p.Arg18Trp | compound heterozygous | 19 | Stargardt Disease |
| 9 | 1015 | c.2453G>A,p.Gly818Glu | c.1804C>T,p.Arg602Trp; c.2828G>A,p.Arg943Gln | compound heterozygous / complex allele | 23 | Stargardt Disease |
| 10 | 1046 | c.2894A>G,p.Asn965Ser | c.2894A>G,p.Asn965Ser | homozygous | 42 | Stargardt Disease |
| 11 | 1128 | c.5324T>A,p.Ile1775Asn | c.5324T>A,p.Ile1775Asn | homozygous | 24 | Stargardt Disease |
| 12 | 1129 | c.2453G>A,p.Gly818Glu | c.3898C>T,p.Arg1300* | compound heterozygous | 25 | Stargardt Disease |
| 13 | 1163 | c.3386G>T,p.Arg1129Leu | c.4139C>T,p.Pro1380Leu | compound heterozygous | 33 | Stargardt Disease |
| 14 | 1175 | c.6221G>T,p.Gly2074Val | c.6282+3A>T | compound heterozygous | 29 | Cone dystrophy |
| 15 | 1388 | c.2828G>A,p.Arg943Gln | c.265G>T,p.Glu89* | compound heterozygous | 41 | Stargardt Disease |
| 16 | 1428 | c.5318C>T,p.Ala1773Val | c.5318C>T,p.Ala1773Val | homozygous | 16 | Stargardt Disease |
| 17 | 1465 | c.6221G>T,p.Gly2074Val | c.2453G>A,p.Gly818Glu | compound heterozygous | 23 | Stargardt Disease |
| 18 | 1871 | c.2453G>A,p.Gly818Glu | c.5824G>C,p.Glu1942Gln; c.6383A>G,p.His2128Arg | compound heterozygous / complex allele | 15 | Stargardt Disease |
| 19 | 1967 | c.723A>T,p.Glu241Asp | c.5114G>A,p.Arg1705Gln | compound heterozygous | 16 | Stargardt Disease |
| 20 | 2008 | c.2453G>A,p.Gly818Glu | c.2453G>A,p.Gly818Glu | homozygous | 20 | Stargardt Disease |
| 21 | 2090 | c.5335T>C,p.Tyr1779His | c.5335T>C,p.Tyr1779His | homozygous | 28 | Stargardt Disease |
| 22 | 2367 | c.4667G>C,p.Arg1556Thr | c.4667G>C,p.Arg1556Thr | homozygous | 15 | Stargardt Disease |
| 23 | 2409 | c.5318C>T,p.Ala1773Val | c.5318C>T,p.Ala1773Val | homozygous | 24 | Stargardt Disease |
| 24 | 2708 | c.2894A>G,p.Asn965Ser | c.4537dup,p.Gln1513Profs*42 | compound heterozygous | 10 | Stargardt Disease |
| 25 | 2719 | c.2453G>A,p.Gly818Glu | c.2453G>A,p.Gly818Glu | homozygous | 33 | Stargardt Disease |
| 26 | 2729 | c.5318C>T,p.Ala1773Val | c.5318C>T,p.Ala1773Val | homozygous | 14 | Stargardt Disease |
| 27 | 2810 | c.1937+1G>A | c.5460+3G>A | compound heterozygous | 23 | Stargardt Disease |
| 28 | 2901 | c.1574T>C,p.Phe525Ser | c.6220G>T,p.Gly2074Val | compound heterozygous | 9 | Stargardt Disease |
| 29 | 3080 | c.5318C>T,p.Ala1773Val | c.5318C>T,p.Ala1773Val | homozygous | 41 | Stargardt Disease |
| 30 | 3286 | c.3383A>G,p.Asp1128Gly | c.4804del,p.Ile1602Tyrfs*8 | compound heterozygous | 45 | Retinitis pigmentosa |
| 31 | 3449 | c.5324T>A,p.Ile1775Asn | c.5512C>G,p.His1838Asp; c.5882G>A,p.Gly1961Glu | compound heterozygous / complex allele | 43 | Stargardt Disease |
| 32 | 3457 | c.6148G>C,p.Val2050Leu | c.6148G>C,p.Val2050Leu | homozygous | 29 | Stargardt Disease |
| 33 | 3478 | c.5318C>T,p.Ala1773Val | c.5318C>T,p.Ala1773Val | homozygous | 11 | Stargardt Disease |
| 34 | 3497 | c.4328G>A,p.Arg1443His | c.2267C>T,p.Ser756Phe; c.2522_2530del,p.Gln841_Met843del | compound heterozygous / complex allele | 17 | Stargardt Disease |
| 35 | 3522 | c.5819T>C,p.Leu1940Pro | c.5324T>A,p.Ile1775Asn | compound heterozygous | 41 | Stargardt Disease |
| 36 | 3529 | c.5318C>T,p.Ala1773Val | c.6221G>T,p.Gly2074Val | compound heterozygous | 14 | Stargardt Disease |
| 37 | 3530 | c.5324T>A,p.Ile1775Asn | c.6446G>C,p.Arg2149Pro | compound heterozygous | 10 | Stargardt Disease |
| 38 | 3566 | c.4919G>A,p.Arg1640Gln | c.4919G>A,p.Arg1640Gln | homozygous | 48 | Retinitis Pigmentosa |
| 39 | 3585 | c.4854G>C,p.Trp1618Cys | c.4919G>A,p.Arg1640Gln | compound heterozygous | 46 | Unspecified macular distrphy |
| 40 | 3612 | c.1804C>T,p.Arg602Trp | c.2453G>A,p.Gly818Glu | compound heterozygous | 22 | Stargardt Disease |
| 41 | 3623 | c.4519G>A,p.Gly1507Arg | c.3898del,p.Arg1300fsAspfs*89 | compound heterozygous | 40 | Stargardt Disease |
| 42 | 3632 | c.1417_1420dup,p.Thr474Asnfs*4 | c.5196+1G>A | compound heterozygous | 11 | Stargardt Disease |
| 43 | 3648 | c.2453G>A,p.Gly818Glu | c.1876_1888del,p.Ala626Leufs*19 | compound heterozygous | 32 | Stargardt Disease |
| 44 | 3702 | c.5318C>T,p.Ala1773Val | c.5318C>T,p.Ala1773Val | homozygous | 22 | Stargardt Disease |
| 45 | 3704 | c.3386G>T,p.Arg1129Leu | c.5318C>T,p.Ala1773Val | compound heterozygous | 23 | Stargardt Disease |
| 46 | 3714 | c.5318C>T,p.Ala1773Val | c.4773G>T,p.Gly1591= | compound heterozygous | 23 | Stargardt Disease |
| 47 | 3758 | c.2453G>A,p.Gly818Glu | c.5318C>T,p.Ala1773Val | compound heterozygous | 20 | Stargardt Disease |
| 48 | 3804 | c.5318C>T,p.Ala1773Val | c.5819T>C,p.Leu1940Pro | compound heterozygous | 26 | Stargardt Disease |
| 49 | 3888 | c.4854G>C,p.Trp1618Cys | c.5318C>T,p.Ala1773Val | compound heterozygous | 15 | Stargardt Disease |
| 50 | 3903 | c.4773G>T,p.Gly1591Gly | c.3814-2A>T | compound heterozygous | 48 | Stargardt Disease |
| 51 | 3912 | c.4978C>T,p.Pro1660Ser | c.5318C>T,p.Ala1773Val | compound heterozygous | 23 | Stargardt Disease |
| 52 | 3917 | c.5819T>C,p.Leu1940Pro | c.6221G>T,p.Gly2074Val | compound heterozygous | 14 | Stargardt Disease |
| 53 | 3923 | c.4919G>A,p.Arg1640Gln | c.4919G>A,p.Arg1640Gln | homozygous | 12 | Stargardt Disease |
| 54 | 3924 | c.5318C>T,p.Ala1773Val | c.5196+1137G>A | compound heterozygous | 48 | Stargardt Disease |
| 55 | 3925 | c.5318C>T,p.Ala1773Val | c.6221G>T,p.Gly2074Val | compound heterozygous | 9 | Stargardt Disease |
| 56 | 3928 | c.5324T>A,p.Ile1775Asn | c.2453G>A,p.Gly818Glu | compound heterozygous | 15 | Stargardt Disease |
| 57 | 3930 | c.5882G>A,p.Gly1961Glu | c.5527C>T,p.Arg1843Trp; c.3113C>T,p.Ala1038Val | compound heterozygous / complex allele | 34 | Stargardt Disease |
| 58 | 3936 | c.4919G>A,p.Arg1640Gln | c.4919G>A,p.Arg1640Gln | homozygous | 13 | Stargardt Disease |
| 59 | 3941 | c.2453G>A,p.Gly818Glu | c.5324T>A,p.Ile1775Asn | compound heterozygous | 37 | Stargardt Disease |
| 60 | 3944 | c.4139C>T,p.Pro1380Leu | c.4667G>C,p.Arg1556Thr | compound heterozygous | 23 | Stargardt Disease |
| 61 | 3945 | c.5318C>T,p.Ala1773Val | c.5318C>T,p.Ala1773Val | homozygous | 32 | Stargardt Disease |
| 62 | 3947 | c.2453G>A,p.Gly818Glu | c.3292C>T,p.Arg1098Cys; c.488_491del,p.Leu163Hisfs*18 | compound heterozygous / complex allele | 23 | Stargardt Disease |
| 63 | 3957 | c.5318C>T,p.Ala1773Val | c.4773G>T,p.Gly1591Gly | compound heterozygous | 26 | Stargardt Disease |
| 64 | 3997 | c.2453G>A,p.Gly818Glu | c.3608-1G>A | compound heterozygous | 21 | Stargardt Disease |
| 65 | 4007 | c.5324T>A,p.Ile1775Asn | c.5324T>A,p.Ile1775Asn | homozygous | 13 | Stargardt Disease |
| 66 | 4019 | c.689G>T,p.Cys230Phe | c.6306C>A,p.Asp2102Glu | compound heterozygous | 11 | Stargardt Disease |
| 67 | 4136 | c.3386G>T,p.Arg1129Leu | c.5318C>T,p.Ala1773Val | compound heterozygous | 12 | Stargardt Disease |
| 68 | 4154 | c.6394G>T,p.Glu2132* | c.2023G>A,p.Val675Ile | compound heterozygous | 25 | Stargardt Disease |
| 69 | 4167 | c.634C>T,p.Arg212Cys | c.5318C>T,p.Ala1773Val | compound heterozygous | 20 | Stargardt Disease |
| 70 | 4213 | c.1804C>T,p.Arg602Trp | c.4313C>A,p.Pro1438Gln | compound heterozygous | 41 | Stargardt Disease |
| 71 | 4214 | c.3292C>T,p.Arg1098Cys | c.2905A>G,p.Lys969Glu; c.488_491del,p.Leu163Hisfs*18 | compound heterozygous / complex allele | 8 | Stargardt Disease |
| 72 | 4220 | c.1222C>T,p.Arg408* | c.1222C>T,p.Arg408* | homozygous | 28 | Retinitis pigmentosa |
| 73 | 4235 | c.3323G>T,p.Arg1108Leu | c.4222T>C,p.Trp1408Arg; c.4918C>T,p.Arg1640Trp | compound heterozygous / complex allele | 8 | Stargardt Disease |
| 74 | 4237 | c.3322C>T,p.Arg1108Cys | c.4222T>C,p.Trp1408Arg; c.4918C>T,p.Arg1640Trp | compound heterozygous / complex allele | 30 | Stargardt Disease |
| 75 | 4260 | c.5318C>T,p.Ala1773Val | c.5318C>T,p.Ala1773Val | homozygous | 27 | Stargardt Disease |
| 76 | 4263 | c.4667G>C,p.Arg1556Thr | c.4139C>T,p.Pro1380Leu | compound heterozygous | 28 | Stargardt Disease |
| 77 | 4289 | c.5318C>T,p.Ala1773Val | c.5318C>T,p.Ala1773Val | homozygous | 3 | Stargardt Disease |
| 78 | 4370 | c.4919G>A,p.Arg1640Gln | c.5318C>T,p.Ala1773Val | compound heterozygous | 14 | Stargardt Disease |
| 79 | 4438 | c.4873C>T,p.His1625Tyr | c.4854G>C,p.Tpr1618Cys | compound heterozygous | 8 | Stargardt Disease |
| 80 | 4447 | c.2453G>A,p.Gly818Glu | c.2894A>G,p.Asn965Ser | compound heterozygous | 41 | Stargardt Disease |
| 81 | 4500 | c.4854G>C,p.Trp1618Cys | c.4854G>C,p.Trp1618Cys | homozygous | 35 | Cone dystrophy |
| 82 | 4529 | c.5318C>T,p.Ala1773Val | c.5318C>T,p.Ala1773Val | homozygous | 18 | Stargardt Disease |
| 83 | 4552 | c.5318C>T,p.Ala1773Val | c.5318C>T,p.Ala1773Val | homozygous | 37 | Stargardt Disease |
| 84 | 4623 | c.4139C>T,p.Pro1380Leu | c.2570T>C,p.Leu857Pro | compound heterozygous | 31 | Stargardt Disease |
| 85 | 4648 | c.2041C>T,p.Arg681* | c.67-1dup | compound heterozygous | 12 | Stargardt Disease |
| 86 | 4677 | c.5318C>T,p.Ala1773Val | c.5318C>T,p.Ala1773Val | homozygous | 28 | Stargardt Disease |
| 87 | 4678 | c.1804C>T,p.Arg602Trp | c.2828G>A,p.Arg943Gln; c.6089G>A,p.Arg2030Gln | compound heterozygous / complex allele | 33 | Stargardt Disease |
| 88 | 4680 | c.5318C>T,p.Ala1773Val | c.5318C>T,p.Ala1773Val | homozygous | 26 | Cone dystrophy |
| 89 | 4681 | c.5318C>T,p.Ala1773Val | c.5318C>T,p.Ala1773Val | homozygous | 20 | Stargardt Disease |
| 90 | 4714 | c.1417_1420dup,p.Thr474Asnfs*4 | c.3386G>T,p.Arg1129Leu | compound heterozygous | 7 | Stargardt Disease |
| 91 | 4720 | c.4328G>A,p.Arg1443His | c.6383A>G,p.His2128Arg | compound heterozygous | 36 | Stargardt Disease |
| 92 | 4724 | c.1937+1G>A | c.5318C>T,p.Ala1773Val | compound heterozygous | 27 | Stargardt Disease |
| 93 | 4739 | c.1798G>T,p.Asp600Tyr | c.1798G>T,p.Asp600Tyr | homozygous | 11 | Stargardt Disease |
| 94 | 4742 | c.5318C>T,p.Ala1773Val | c.4854G>C,p.Trp1618Cys | compound heterozygous | 37 | Stargardt Disease |
| 95 | 4745 | c.5318C>T,p.Ala1773Val | c.4854G>C,p.Trp1618Cys | compound heterozygous | 33 | Stargardt Disease |
| 96 | 4751 | c.4667G>C,p.Arg1556Thr | c.6306C>A,p.Asp2102Glu | compound heterozygous | 17 | Stargardt Disease |
| 97 | 4753 | c.634C>T,p.Arg212Cys | c.5951T>G,p.Met1984Arg | compound heterozygous | 25 | Stargardt Disease |
| 98 | 4764 | c.5324T>A,p.Ile1775Asn | c.6221G>T,p.Gly2074Val | compound heterozygous | 16 | Stargardt Disease |
| 99 | 4789 | c.2453G>A,p.Gly818Glu | c.3814-2A>T | compound heterozygous | 37 | Stargardt Disease |
| 100 | 4798 | c.5413A>G,p.Asn1805Asp | c.4243dup,p.Thr1415Asnfs*7 | compound heterozygous | 23 | Stargardt Disease |
| 101 | 4811 | c.3386G>T,p.Arg1129Leu | c.5318C>T,p.Ala1773Val | compound heterozygous | 22 | Stargardt Disease |
| 102 | 4874 | c.2453G>A,p.Gly818Glu | c.6119G>A,p.Arg2040Gln | compound heterozygous | 44 | Non-specified macular dystrophy |
| 103 | 4876 | c.5318C>T,p.Ala1773Val | c.2807del,p.Lys936Argfs*14 | compound heterozygous | 18 | Stargardt Disease |
| 104 | 4877 | c.3352C>T,p.His1118Tyr | c.3352C>T,p.His1118Tyr | homozygous | 21 | Stargardt Disease |
| 105 | 4935 | c.2396C>T,p.Pro799Leu | c.4919G>A,p.Arg1640Gln | compound heterozygous | 48 | Stargardt Disease |
| 106 | 4939 | c.4519G>A,p.Gly1507Arg | c.6221G>T,p.Gly2074Val | compound heterozygous | 59 | Stargardt Disease |
| 107 | 4958 | c.4070C>A,p.Ala1357Glu | c.4070C>A,p.Ala1357Glu | homozygous | 35 | Retinitis Pigmentosa |
| 108 | 4964 | c.4436G>A,p.Trp1479* | c.2453G>A,p.Gly818Glu | compound heterozygous | 27 | Stargardt Disease |
| 109 | 4965 | c.4436G>A,p.Trp1479* | c.4436G>A,p.Trp1479* | homozygous | 70 | Retinitis Pigmentosa |
| 110 | 5002 | c.4854G>C,p.Trp1618Cys | c.634C>T,p.Arg212Cys | compound heterozygous | 30 | Stargardt Disease |
| 111 | 5049 | c.4926C>G,p.Ser1642Arg; c.5044_5058del,p.Val1682_1686del | c.4926C>G,p.Ser1642Arg; c.5044_5058del,p.Val1682_1686del | homozygous / complex alleles (uniparental disomy) | 20 | Stargardt Disease |
| 112 | 5066 | c.5318C>T,p.Ala1773Val | c.5318C>T,p.Ala1773Val | homozygous | 18 | Cone dystrophy |
| 113 | 5113 | c.4519G>A,p.Gly1507Arg | c.4852T>C,p.Trp1618Arg | compound heterozygous | 54 | Stargardt Disease |
| 114 | 5122 | c.1804C>T,p.Arg602Trp | c.1804C>T,p.Arg602Trp | compound heterozygous | 13 | Stargardt Disease |
| 115 | 5123 | c.2453G>A,p.Gly818Glu | c.3056C>T,p.Thr1019Met | compound heterozygous | 22 | Stargardt Disease |
| 116 | 5188 | c.1804C>T,p.Arg602Trp | c.5318C>T,p.Ala1773Val | compound heterozygous | 31 | Stargardt Disease |
| 117 | 5216 | c.5461-10T>C | c.2588G>C,p.Gly863Ala | compound heterozygous | 41 | Stargardt Disease |
| 118 | 5262 | c.6306C>A,p.Asp2102Glu | c.4352+61G>A | compound heterozygous | 69 | Stargardt Disease |
| 119 | 5329 | c.5318C>T,p.Ala1773Val | c.5318C>T,p.Ala1773Val | homozygous | 25 | Stargardt Disease |
| 120 | 5358 | c.287A>T, p.Asn96Ile | c.5318C>T, p.Ala1773Val | compound heterozygous | 27 | Stargardt Disease |
| 121 | 5367 | c.3322C>T,p.Arg1108Cys | c.3386G>T,p.Arg1129Leu | compound heterozygous | 53 | Stargardt Disease |
| 122 | 5375 | c.3210_3211dup,p.Ser1071Cysfs*14 | c.5318C>T,p.Ala1773Val | compound heterozygous | 29 | Stargardt Disease |
| 123 | 5388 | c.3113C>T,p.Ala1038Val | c.4919G>A,p.Arg1640Gln | compound heterozygous | 20 | Stargardt Disease |
| 124 | 5417 | c.1766G>A,p.Trp589* | c.2453G>A,p.Gly818Glu | compound heterozygous | 26 | Non-specified macular dystrophy |
| 125 | 5441 | c.2041C>T,p.Arg681* | c.2570T>C,p.Leu857Pro | compound heterozygous | 11 | Stargardt Disease |
| 126 | 5483 | c.3386G>T,p.Arg1129Leu | c.634C>T,p.Arg212Cys | compound heterozygous | 57 | Stargardt Disease |
| 127 | 5509 | c.3386G>T,p.Arg1129Leu | c.4854G>C,p.Trp1618Cys | compound heterozygous | 9 | Stargardt Disease |
| 128 | 5530 | c.5951T>G,p.Met1984Arg | c.6221G>T,p.Gly2074Val | compound heterozygous | 15 | Stargardt Disease |
| 129 | 5535 | c.6221G>T,p.Gly2074Val | c.2453G>A,p.Gly818Glu | compound heterozygous | 19 | Stargardt Disease |
| 130 | 5574 | c.2453G>A,p.Gly818Glu | c.4457C>T,p.Pro1486Leu | compound heterozygous | 58 | Stargardt Disease |
| 131 | 5595 | c.5318C>T,p.Ala1773Val; | c.3113C>T,p.Ala1038Val; c.5882G>A,p.Gly1961Glu | compound heterozygous / complex allele | 42 | Stargardt Disease |
| 132 | 5624 | c.5318C>T,p.Ala1773Val | c.5318C>T,p.Ala1773Val | homozygous | 9 | Stargardt Disease |
| 133 | 5626 | c.6221G>T,p.Gly2074Val | c.3308T>G,p.Leu1103Arg | compound heterozygous | 11 | Stargardt Disease |
| 134 | 5655 | c.5318C>T,p.Ala1773Val | c.6094C>T,p.His2032Tyr | compound heterozygous | 29 | Stargardt Disease |
| 135 | 5689 | c.2894A>G,p.Asn965Ser | c.5318C>T,p.Ala1773Val | compound heterozygous | 17 | Stargardt Disease |
| 136 | 5694 | c.3386G>T,p.Arg119Leu | c.4926C>G,p.Ser1642Arg; c.5044_5058del,p.Val1682_1686del | compound heterozygous / complex allele | 32 | Stargardt Disease |
| 137 | 5740 | c.4139C>T,p.Pro1380Leu | c.4519G>A,p.Gly1507Arg | compound heterozygous | 41 | Stargardt Disease |
| 138 | 5769 | c.5498T>G,p.Leu1833Arg | c.1648G>A,p.Gly550Arg | compound heterozygous | 39 | Stargardt Disease |
| 139 | 5771 | c.1222C>T,p.Arg408* | c.634C>T,p.Arg212Cys | compound heterozygous | 13 | Stargardt Disease |
| 140 | 5772 | c.1804C>T,p.Arg602Trp | c.4849-1G>A | compound heterozygous | 30 | Stargardt Disease |
| 141 | 5777 | c.2453G>A,p.Gly818Glu | c.4854G>c,p.Trp1618Cys | compound heterozygous | 22 | Stargardt Disease |
| 142 | 5792 | c.2453G>A,p.Gly818Glu | c.4919G>A,p.Arg1640Gln | compound heterozygous | 38 | Stargardt Disease |
| 143 | 5795 | c.4919G>A,p.Arg1640Gln | c.5318C>T,p.Ala1773Val | compound heterozygous | 35 | Stargardt Disease |
| 144 | 5797 | c.6221G>T,p.Gly2074Val | c.5882G>A,p.Gly1961Glu | compound heterozygous | 18 | Stargardt Disease |
| 145 | 5905 | c.3386G>T,p.Arg1129Leu | c.438del, p.Ile146Metfs8 | compound heterozygous | 24 | Non specifid macular dystrophy |
| 146 | 5927 | c.5318C>T,p.Ala1773Val | c.5318C>T,p.Ala1773Val | homozygous | 49 | Cone dystrophy |
| 147 | 5929 | c.2453G>A,p.Gly818Glu | c.2453G>A,p.Gly818Glu | homozygous | 81 | Stargardt Disease |
| 148 | AP-1 | c.4773G>T,p.Gly1591Gly | c.5318C>T,p.Ala1773Val | compound heterozygous | 23 | Stargardt Disease |
| 149 | AP-2 | c.3056C>T,p.Thr1019Met | c.3210_3211dup,p.Ser1071Cysfs*14 | compound heterozygous | 19 | Stargardt Disease |
| 150 | AP-3 | c.5113C>T,p.Arg1705Trp | c.5113C>T,p.Arg1705Trp | homozygous | 28 | Stargardt Disease |
| 151 | AP-4 | c.3386G>T,p.Arg1129Leu | c.4854G>C,p.Trp1618Cys | compound heterozygous | 27 | Stargardt Disease |
| 152 | AP-5 | c.5318C>T,p.Ala1773Val | c.5318C>T,p.Ala1773Val | homozygous | 18 | Stargardt Disease |
| 153 | AP-6 | c.4667G>C,p.Arg1556Thr | c.5714+5G>A | compound heterozygous | 36 | Stargardt Disease |
| 154 | AP-7 | c.4457C>T,p.Pro1486Leu | c.4854G>C,p.Trp1618Cys | compound heterozygous | 39 | Stargardt Disease |
| 155 | AP-8 | c.4519G>A,p.Gly1507Arg | c.6306C>A,p.Asp2102Glu | compound heterozygous | 53 | Stargardt Disease |
| 156 | AP-9 | c.3898C>T,p.Arg1300* | c.6446G>C,p.Arg2149Pro | compound heterozygous | 11 | Stargardt Disease |
| 157 | AP-10 | c.3113C>T,p.Ala1038Val | c.4558G>C,p.Glu1520Gln | compound heterozygous | 59 | Stargardt Disease |
| 158 | AP-11 | c.4919G>A,p.Arg1640Gln | c.4919G>A,p.Arg1640Gln | homozygous | 10 | Non-specified macular dystrophy |
| 159 | AP-12 | c.5324T>A,p.Ile1775Asn | c.6089G>A,p.Arg2030Gln | compound heterozygous | 33 | Cone dystrophy |
| 160 | AP-13 | c.4854G>C,p.Trp1618Cys | c.4854G>C,p.Trp1618Cys | homozygous | 14 | Stargardt Disease |
| 161 | AP-14 | c.4854G>C,p.Trp1618Cys | c.5714+5G>A | compound heterozygous | 26 | Stargardt Disease |
| 162 | EC-100 | c.3602T>G,p.Leu1201Arg | c.5196+1137G>A | compound heterozygous | 56 | Stargardt Disease |
| 163 | EC-103 | c.4457C>T,p.Pro1486Leu | c.5196+1G>A | compound heterozygous | 33 | Stargardt Disease |
| 164 | EC-106 | c.4793C>A,p.Ala1598Asp | whole deletion exon 7 | compound heterozygous | 46 | Retinitis pigmentosa |
| 165 | EC-169 | c.2453G>A,p.Gly818Glu | c.4519G>A,p.Gly1507Arg | compound heterozygous | 49 | Stargardt Disease |
| 166 | EC-200 | c.634C>T,p.Arg212Cys | c.6221G>T,p.Gly2074Val | compound heterozygous | 6 | Stargardt Disease |
| 167 | EC-204 | c.2453G>A,p.Gly818Glu | c.5824G>C,p.Glu1942Gln | compound heterozygous | 52 | Stargardt Disease |
| 168 | EC-40 | c.2453G>A,p.Gly818Glu | c.4457C>T,p.Pro1486Leu | compound heterozygous | 53 | Stargardt Disease |
| 169 | EC-46 | c.2041C>T,p.Arg681* | c.3056C>T,p.Thr1019Met | compound heterozygous | 14 | Stargardt Disease |
| 170 | EC-52 | c.4457C>T,p.Pro1486Leu | c.4457C>T,p.Pro1486Leu | homozygous | 38 | Stargardt Disease |
| 171 | EC-62 | c.1819G>C,p.Gly607Arg | c.6401A>G,p.Glu2134Gly | compound heterozygous | 17 | Stargardt Disease |
| 172 | EC-64 | c.6686T>C,p.Leu2229Pro | c.6686T>C,p.Leu2229Pro | homozygous | 64 | Stargardt Disease |
| 173 | EC-69 | c.2453G>A,p.Gly818Glu | c.5318C>T,p.Ala1773Val | compound heterozygous | 22 | Stargardt Disease |
| 174 | EC-71 | c.2888delG,p.Gly963Alafs*14 | c.6339C>G,p.Ile2113Met | compound heterozygous | 11 | Stargardt Disease |
| 175 | EC-75 | c.2905A>G,p.Lys969Glu | c.2453G>C,p.Gly818Ala | compound heterozygous | 15 | Stargardt Disease |
| 176 | EC-76 | c.4457C>T,p.Pro1486Leu | c.4854G>C,p.Trp1618Cys | compound heterozygous | 41 | Stargardt Disease |
| 177 | EC-153 | c.3056C>T,p.Thr1019Met | c.634C>T,p.Arg212Cys | compound heterozygous | 10 | Cone dystrophy |
| 178 | EC-221 | c.3898C>T, p.Arg1300* | c.3898C>T, p.Arg1300* | homozygous | 41 | Cone dystrophy |
| 179 | XT-1 | c.5318C>T,p.Ala1773Val | c.634C>T,p.Arg212Cys | compound heterozygous | 23 | Stargardt Disease |
| 180 | XT-2 | c.1804C>T,p.Arg602Trp | c.3386G>T,p.Arg1129Leu | compound heterozygous | 39 | Stargardt Disease |
| 181 | XT-3 | c.2908del,p.Thr970Profs*7 | c.5882G>A,p.Gly1961Glu | compound heterozygous | 24 | Stargardt Disease |
| 182 | XT-4 | c.3386G>T,p.Arg1129Leu | c.4139C>T,p.Pro1380Leu | compound heterozygous | 44 | Stargardt Disease |
| 183 | XT-5 | c.4537dup,p.Gln1513Profs*42 | c.5461-1G>T | compound heterozygous | 52 | Stargardt Disease |
| 184 | XT-6 | c.3386G>T,p.Arg1129Leu | c.4457C>T,p.Pro1486Leu | compound heterozygous | 31 | Stargardt Disease |
| 185 | XT-7 | c.3386G>T,p.Arg1129Leu | c.6718A>G,p.Thr2240Ala; c.4352+61G>A | compound heterozygous / complex allele | 31 | Stargardt Disease |
| 186 | XT-8 | c.2894A>G,p.Asn965Ser | c.5196+1137G>A | compound heterozygous | 54 | Stargardt Disease |
| 187 | XT-9 | c.5318C>T,p.Ala1773Val | c.6221G>T,p.Gly2074Val | compound heterozygous | 17 | Stargardt Disease |
| 188 | XT-10 | c.5318C>T,p.Ala1773Val | c.4926C>G,p.Ser1642Arg; c.5044_5058del,p.Val1682_1686del | compound heterozygous / complex alleles | 36 | Stargardt Disease |
| 189 | XT-11 | c.4854G>C,p.Trp1618Cys | c.4854G>C,p.Trp1618Cys | homozygous | 62 | Stargardt Disease |
| 190 | XT-12 | c.3113C>T,p.Ala1038Val | c.4919G>A,p.Arg1640Gln | compound heterozygous | 57 | Stargardt Disease |
| 191 | XT-13 | c.6320G>A,p.Arg2107His | c.5333T>A,p.Met1778Lys | compound heterozygous | 33 | Stargardt Disease |
| 192 | XT-14 | c.3386G>T,p.Arg1129Leu | c.4253+4C>T; c.6718A>G,p.Thr2240Ala | compound heterozygous / complex allele | 37 | Stargardt Disease |
| 193 | HL-1 | c.723A>T,p.Glu241Asp | c.1994del,p.Tyr665Serfs*5 | compound heterozygous | 2 | Stargardt Disease |
| 194 | HL-2 | c.634C>T,p.Arg212Cys | c.735T>G,p.Tyr245* | compound heterozygous | 22 | Stargardt Disease |
| 195 | HL-3 | c.3056C>T,p.Thr1019Met | c.634C>T,p.Arg212Cys | compound heterozygous | 35 | Stargardt Disease |
| 196 | HL-4 | c.4253+4C>T | c.5318C>T,p.Ala1773Val | compound heterozygous | 15 | Stargardt Disease |
| 197 | HL-5 | c.4854G>C,p.Trp1618Cys | c.4854G>C,p.Trp1618Cys | homozygous | 15 | Stargardt Disease |
| 198 | HL-6 | c.3210_3211dup,p.Ser1071Cysfs*14 | c.3210_3211dup,p.Ser1071Cysfs*14 | homozygous | 3 | Stargardt Disease |
| 199 | HL-7 | c.4919G>A,p.Arg1640Gln | c.4919G>A,p.Arg1640Gln | homozygous | 13 | Stargardt Disease |
| 200 | HL-8 | c.5324T>A,p.Ile1775Asn | c.6221G>T,p.Gly2074Val | compound heterozygous | 13 | Stargardt Disease |
| 201 | HL-9 | c.3386G>T,p.Arg1129Leu | c.5324T>A,p.Ile1775Asn | compound heterozygous | 41 | Stargardt Disease |
| 202 | HL-10 | c.5318C>T,p.Ala1773Val | c.5318C>T,p.Ala1773Val | homozygous | 11 | Stargardt Disease |
| 203 | HL-11 | c.179C>T,p.Ala60Val | c.4667G>C,p.Arg1556Thr | compound heterozygous | 17 | Stargardt Disease |
| 204 | HL-12 | c.634C>T,p.Arg212Cys | c.5882G>A,p.Gly1961Glu | compound heterozygous | 18 | Stargardt Disease |
| 205 | INV-1 | c.3386G>T,p.Arg1129Leu | c.4139C>T,p.Pro1380Leu | compound heterozygous | 37 | Stargardt Disease |
| 206 | NA222 | c.2297_2299del,p.Gly766del | c.6397T>C,p.Cys2133Arg | compound heterozygous | 34 | Stargardt Disease |
| 207 | NA225 | c.4577C>T,p.Thr1526Met | c.5318C>T,p.Ala1773Val | compound heterozygous | 52 | Stargardt Disease |
| 208 | SD10 | c.5318C>T,p.Ala1773Val | c.6308C>A,p.Pro2103His; c.6299G>A,p.Gly2100Glu | compound heterozygous / complex allele | 10 | Stargardt Disease |
| 209 | SD20 | c.5714+5G>A | c.2453G>A,p.Gly818Glu | compound heterozygous | 46 | Stargardt Disease |
| 210 | SD44 | c.4577C>T,p.Thr1526Met | deletion exons 17-23 | compound heterozygous | 10 | Cone dystrophy |
| 211 | SD62 | c.5318C>T,p.Ala1773Val | c.2741_2742del,p.His914Argfs*5 | compound heterozygous | 27 | Non-specified macular dystrophy |
