# Supplementary material for: Genotypic spectrum of ABCA4-associated retinal degenerations in 211 unrelated Mexican patients: identification of 22 novel disease-causing variants
Source: Mol Genet Genomics. 2024 Aug 20;299(1):79. doi: 10.1007/s00438-024-02174-x (PMC11335775; doi:10.1007/s00438-024-02174-x)
Supplement: Supplementary file 2 — Supplementary Material 2 [file 438_2024_2174_MOESM2_ESM.docx]

**Supplementary table 2**. *ABCA4* pathogenic/likely pathogenic variants identified in a cohort of 211 Mexican individuals with retinal dystrophies.

| **Variant**  **#** | **Localization**  **(E:exon; I:intron)** | **Nucleotide variant (cDNA)** | **Protein Variant** | **Number of alleles** | **Type of variant** | **Allele frequency in gnomAD** |
| --- | --- | --- | --- | --- | --- | --- |
| **1** | E1 | c.52C>T | p.Arg18Trp | 1 | missense | 0.0016% |
| **2** | I2 | c.67-1dup |  | 1 | null variant (acceptor site) | NF |
| **3** | E3 | c.179C>T | p.Ala60Val | 1 | missense | 0.0016% |
| **4** | E3 | c.265G>T | p.Glu89* | 1 | null variant (stop gain) | NF |
| **5** | E3 | c.287A>T | p.Asn96Ile | 1 | missense | NF |
| **6** | E4 | c.438del | p.Ile146Metfs8 | 1 | null variant (frameshift indel) | NF |
| **7** | E5 | c.488_491del | p.Leu163Hisfs*18 | 2 | null variant (frameshift indel) | 0.0004% |
| **8** | E6 | c.634C>T | p.Arg212Cys | 12 | missense | 0.0107% |
| **9** | E6 | c.689G>T | p.Cys230Phe | 1 | missense | NF |
| **10** | E6 | c.723A>T | p.Glu241Asp | 2 | missense | NF |
| **11** | E6 | c.735T>G | p.Tyr245* | 1 | null variant (stop gain) | NF |
| **12** | I6 | c.768+1G>A |  | 1 | null variant (donor site) | NF |
| **13** | E7 | Whole deletion |  | 1 | CNV | NF |
| **14** | E8 | c.868C>T | p.Arg290Trp | 1 | missense | 0.002% |
| **15** | E9 | c.1222C>T | p.Arg408* | 3 | null variant (stop gain) | 0.0018% |
| **16** | E11 | c.1417_1420dup | p.Thr474Asnfs*4 | 2 | null variant (frameshift indel) | NF |
| **17** | E12 | c.1574T>C | p.Phe525Ser | 1 | missense | NF |
| **18** | E12 | c.1648G>A | p.Gly550Arg | 1 | missense | 0.0004% |
| **19** | E13 | c.1766G>A | p.Trp589* | 1 | null variant (stop gain) | NF |
| **20** | E13 | c.1798G>T | p.Asp600Tyr | 2 | missense | 0.0004% |
| **21** | E13 | c.1804C>T | p.Arg602Trp | 9 | missense | 0.0044% |
| **22** | E13 | c.1819G>C | p.Gly607Arg | 1 | missense | NF |
| **23** | E13 | c.1876_1888del | p.Ala626Leufs*19 | 1 | null variant (frmeshift indel) | NF |
| **24** | I13 | c.1937+1G>A | ---- | 2 | null variant (donor site) | 0.0032% |
| **25** | E14 | c.1994del | p.Tyr665Serfs*5 | 1 | null variant (frameshift indel) | NF |
| **26** | E14 | c.2023G>A | p.Val675Ile | 1 | missense | 0.0076% |
| **27** | E14 | c.2041C>T | p.Arg681* | 3 | null variant (stop gain) | 0.0008% |
| **28** | E15 | c.2267C>T | p.Ser756Phe | 1 | missense | 0.006% |
| **29** | E15 | c.2297_2299del | p.Gly766del | 1 | deletion | NF |
| **30** | E16 | c.2396C>T | p.Pro799Leu | 1 | missense | 0.0057% |
| **31** | E16 | c.2453G>A | p.Gly818Glu | 32 | missense | 0.0191% |
| **32** | E16 | c.2453G>C | p.Gly818Ala | 1 | missense | NF |
| **33** | E16 | c.2522_2530del | p.Gln841_Met843del | 1 | deletion | NF |
| **34** | E16 | c.2570T>C | p.Leu857Pro | 2 | missense | 0.0004% |
| **35** | E17 | c.2588G>C | p.Gly863Ala | 1 | missense | 0.4295% |
| **36** | E17-23 | deletion | ---- | 1 | CNV | NF |
| **37** | E18 | c.2741_2742del | p.His914Argfs*5 | 1 | null variant (frameshift indel) | NF |
| **38** | E19 | c.2807del | p.Lys936Argfs*14 | 1 | null variant (frameshift indel) | NF |
| **39** | E19 | c.2828G>A | p.Arg943Gln | 3 | missense | 3.0122% |
| **40** | E19 | c.2888delG | p.Gly963Alafs*14 | 1 | null variant (frameshift indel) | 0.0004% |
| **41** | E19 | c.2894A>G | p.Asn965Ser | 6 | missense | 0.0135% |
| **42** | E19 | c.2905A>G | p.Lys969Glu | 2 | missense | NF |
| **43** | E19 | c.2908del | p.Thr970Profs*7 | 1 | null variant (frameshift indel) | NF |
| **44** | E20 | c.3041T>G | p.Leu1014Arg | 1 | missense | NF |
| **45** | E21 | c.3056C>T | p.Thr1019Met | 5 | missense | 0.0032% |
| **46** | E21 | c.3113C>T | p.Ala1038Val | 5 | missense | 0.1755% |
| **47** | E22 | c.3210_3211dup | p.Ser1071Cysfs*14 | 4 | null variant (frameshift indel) | 0.002% |
| **48** | E22 | c.3292C>T | p.Arg1098Cys | 2 | missense | 0.0024% |
| **49** | E22 | c.3308T>G | p.Leu1103Arg | 1 | missense | NF |
| **50** | E22 | c.3322C>T | p.Arg1108Cys | 2 | missense | 0.0127% |
| **51** | E22 | c.3323G>T | p.Arg1108Leu | 1 | missense | NF |
| **52** | E23 | c.3352C>T | p.His1118Tyr | 2 | missense | NF |
| **53** | E23 | c.3383A>G | p.Asp1128Gly | 1 | missense | NF |
| **54** | E23 | c.3386G>T | p.Arg1129Leu | 19 | missense | 0.0297% |
| **55** | E24 | c.3602T>G | p.Leu1201Arg | 1 | missense | 0.9019% |
| **56** | I25 | c.3608-1G>A | ---- | 1 | null variant (acceptor site) | NF |
| **57** | I26 | c.3814-2A>T | ---- | 2 | null variant (acceptor site) | NF |
| **58** | E27 | c.3898C>T | p.Arg1300* | 4 | null variant (stop gain) | 0.0016% |
| **59** | E27 | c.3898del | p.Arg1300fsAspfs*89 | 1 | null variant (frameshift indel) | NF |
| **60** | E27 | c.4070C>A | p.Ala1357Glu | 2 | missense | NF |
| **61** | E28 | c.4139C>T | p.Pro1380Leu | 7 | missense | 0.0234% |
| **62** | E28 | c.4222T>C | p.Trp1408Arg | 2 | missense | 0.0016% |
| **63** | E28 | c.4243dup | p.Thr1415Asnfs*7 | 1 | null variant (frameshift indel) | NF |
| **64** | E28 | c.4249_4251del | p.Phe1417del | 1 | deletion | NF |
| **65** | I28 | c.4253+4C>T | ---- | 2 | null variant (splicing) | 0.0024% |
| **66** | E29 | c.4313C>A | p.Pro1438Gln | 1 | missense | 0.0004% |
| **67** | E29 | c.4328G>A | p.Arg1443His | 3 | missense | 0.0021% |
| **68** | I29 | c.4352+61G>A |  | 2 | deep intronic | NF |
| **69** | E30 | c.4436G>A | p.Trp1479* | 3 | null variant (stop gain) | NF |
| **70** | E30 | c.4457C>T | p.Pro1486Leu | 8 | missense | 0.0122% |
| **71** | E30 | c.4519G>A | p.Gly1507Arg | 6 | missense | 0.0157% |
| **72** | E30 | c.4537dup | p.Gln1513Profs*42 | 2 | null variant (frameshift indel) | NF |
| **73** | E31 | c.4558G>C | p.Glu1520Gln | 1 | missense | NF |
| **74** | E31 | c.4577C>T | p.Thr1526Met | 2 | missense | 0.0064% |
| **75** | E32 | c.4667G>C | p.Arg1556Thr | 7 | missense | 0.0008% |
| **76** | E33 | c.4773G>T | p.Gly1591= | 4 | missense | 0.0028% |
| **77** | E34 | c.4793C>A | p.Ala1598Asp | 1 | missense | 0.0024% |
| **78** | E34 | c.4804del | p.Ile1602Tyrfs*8 | 1 | null variant (frameshift indel) | NF |
| **79** | I35 | c.4849-1G>A | ---- | 1 | null variant (acceptor site) | 0.0024% |
| **80** | E35 | c.4852T>C | p.Trp161rg | 1 | missense | NF |
| **81** | E35 | c.4854G>C | p.Trp1618Cys | 20 | missense | 0.0016% |
| **82** | E35 | c.4873C>T | p.His1625Tyr | 1 | missense | NF |
| **83** | E35 | c.4880del | p.Leu1627Argfs*35 | 1 | null variant (frameshift indel) | 0.0016% |
| **84** | E35 | c.4918C>T | p.Arg1640Trp | 2 | missense | 0.0032% |
| **85** | E35 | c.4919G>A | p.Arg1640Gln | 17 | missense | 0.0032% |
| **86** | E35 | c.4926C>G | p.Ser1642Arg | 4 | missense | 0.0042% |
| **87** | E35 | c.4978C>T | p.Pro1660Ser | 1 | missense | 0.0008% |
| **88** | E36 | c.5044_5058del | p.Val1682_Val1686del | 5 | deletion | 0.0028% |
| **89** | E36 | c.5113C>T | p.Arg1705Trp | 2 | missense | 0.0011% |
| **90** | E36 | c.5114G>A | p.Arg1705Gln | 1 | missense | 0.0103% |
| **91** | I36 | c.5196+1G>A | ---- | 2 | null variant (donor site) | 0.0022% |
| **92** | I36 | c.5196+1137G>A | ----- | 3 | deep intronic | 0.0096% |
| **93** | E38 | c.5318C>T | p.Ala1773Val | 79 | missense | 0.0076% |
| **94** | E38 | c.5324T>A | p.Ile1775Asn | 13 | missense | 0.0008% |
| **95** | E38 | c.5333T>A | p.Met1778Lys | 1 | missense | NF |
| **96** | E38 | c.5335T>C | p.Tyr1779His | 2 | missense | NF |
| **97** | E38 | c.5413A>G | p.Asn1805Asp | 1 | missense | 0.0032% |
| **98** | I38 | c.5460+3G>A | ---- | 1 | null variant (splicing) | 0.008% |
| **99** | I38 | c.5461-10T>C | ---- | 1 | null variant (splicing) | 0.022% |
| **100** | I38 | c.5461-1G>T | ---- | 1 | null variant (acceptor site) | NF |
| **101** | E39 | c.5498T>G | p.Leu1833Arg | 1 | missense | NF |
| **102** | E39 | c.5512C>G | p.His1838Asp | 1 | missense | 0.0004% |
| **103** | E39 | c.5527C>T | p.Arg1843Trp | 1 | missense | 0.0016% |
| **104** | I40 | c.5714+5G>A | ----- | 3 | null variant (splicing) | 0.0297% |
| **105** | E41 | c.5819T>C | p.Leu1940Pro | 3 | missense | 0.0021% |
| **106** | E41 | c.5824G>C | p.Glu1942Gln | 2 | missense | 0.0032% |
| **107** | E42 | c.5882G>A | p.Gly1961Glu | 7 | missense | 0.4564% |
| **108** | E43 | c.5951T>G | p.Met1984Arg | 2 | missense | 0.0004% |
| **109** | E44 | c.6089G>A | p.Arg2030Gln | 2 | missense | 0.0354% |
| **110** | E44 | c.6094C>T | p.His2032Tyr | 1 | missense | NF |
| **111** | E44 | c.6119G>A | p.Arg2040Gln | 1 | missense | 0.0311% |
| **112** | E45 | c.6220G>T | p.Gly2074Cys | 1 | missense | NF |
| **113** | E45 | c.6221G>T | p.Gly2074Val | 14 | missense | 0.0036% |
| **114** | E45 | c.6148G>C | p.Val2050Leu | 2 | missense | 0.2857% |
| **115** | I45 | c.6282+3A>T | ---- | 1 | null variant (splicing) | NF |
| **116** | E46 | c.6299G>A | p.Gly2100Glu | 1 | missense | NF |
| **117** | E46 | c.6306C>A | p.Asp2102Glu | 4 | missense | 0.0028% |
| **118** | E46 | c.6308C>A | p.Pro2103His | 1 | missense | NF |
| **119** | E46 | c.6320G>A | p.Arg2107His | 1 | missense | 0.2027% |
| **120** | E46 | c.6339C>G | p.Ile2113Met | 1 | missense | NF |
| **121** | E46 | c.6383A>G | p.His2128Arg | 2 | missense | 0.0008% |
| **122** | E47 | c.6394G>T | p.Glu2132* | 1 | null variant (stop gain) | NF |
| **123** | E47 | c.6397T>C | p.Cys2133Arg | 1 | missense | NF |
| **124** | E47 | c.6401A>G | p.Glu2134Gly | 1 | missense | NF |
| **125** | E47 | c.6446G>C | p.Arg2149Pro | 2 | missense | NF |
| **126** | E48 | c.6686T>C | p.Leu2229Pro | 2 | missense | 0.0068% |
| **127** | E48 | c.6718A>G | p.Thr2240Ala | 2 | missense | 0.0025% |

NF: variant not found in gnomAD
