# Supplementary material for: Genotypic spectrum of ABCA4-associated retinal degenerations in 211 unrelated Mexican patients: identification of 22 novel disease-causing variants
Source: Mol Genet Genomics. 2024 Aug 20;299(1):79. doi: 10.1007/s00438-024-02174-x (PMC11335775; doi:10.1007/s00438-024-02174-x)
Supplement: Supplementary file 3 — Supplementary Material 3 [file 438_2024_2174_MOESM3_ESM.docx]

| **Sample**  **#** | **Variants identified** | **Allele 1** | **Allele 2** |
| --- | --- | --- | --- |
| 1015 | c.1804C>T,p.Arg602Trp;  c.2828G>A,p.Arg943Gln;  c.2453G>A,p.Gly818Glu | c.2453G>A (p.Gly818Glu) | c.1804C>T,p.Arg602Trp; c.2828G>A,p.Arg943Gln; |
| 1871 | c.2453G>A,p.Gly818Glu;  c.5824G>C,p.Glu1942Gln;  c.6383A>G,p.His2128Arg | c.2453G>A (p.Gly818Glu) | c.5824G>C,p.Glu1942Gln; c.6383A>G,p.His2128Arg |
| 3449 | c.5512C>G,p.His1838Asp;  c.5882G>A,p.Gly1961Glu;  c.5324T>A,p.Ile1775Asn | c.5324T>A (p.Ile1775Asn) | c.5512C>G,p.His1838Asp; c.5882G>A,p.Gly1961Glu |
| 3497 | c.4328G>A,p.Arg1443His;  c.2267C>T,p.Ser756Phe;  c.2522_2530del,p.Gln841_Met843del | c.4328G>A (p.Arg1443His) | c.2267C>T,p.Ser756Phe; c.2522_2530del,p.Gln841_Met843del |
| 3930* | c.5882G>A,p.Gly1961Glu;  c.5527C>T,p.Arg1843Trp;  c.3113C>T,p.Ala1038Val | c.5882G>A (p.Gly1961Glu) | c.5527C>T (p.Arg1843Trp);  c.3113C>T (p.Ala1038Val) |
| 3947 | c.2453G>A,p.Gly818Glu;  c.3292C>T,p.Arg1098Cys;  c.488_491del,p.Leu163Hisfs*18 | c.2453G>A, (p.Gly818Glu) | c.3292C>T (p.Arg1098Cys); c.488_491del (p.Leu163Hisfs*18) |
| 4214* | c.488_491del,p.Leu163Hisfs*18;  c.3292C>T,p.Arg1098Cys;  c.2905A>G,p.Lys969Glu | c.2905A>G (p.Lys969Glu) | c.3292C>T (p.Arg1098Cys); c.488_491del (p.Leu163Hisfs*18) |
| 4235 | c.4222T>C,p.Trp1408Arg;  c.4918C>T,p.Arg1640Trp;  c.3323G>T,p.Arg1108Leu | c.3323G>T (p.Arg1108Leu) | c.4222T>C (p.Trp1408Arg);  c.4918C>T (p.Arg1640Trp) |
| 4237 | c.3322C>T,p.Arg1108Cys;c.4222T>C,p.Trp1408Arg; c.4918C>T,p.Arg1640Trp | c.3322C>T (p.Arg1108Cys) | c.4222T>C (p.Trp1408Arg); c.4918C>T (p.Arg1640Trp) |
| 4678* | c.1804C>T,p.Arg602Trp; c.2828G>A,p.Arg943Gln; c.6089G>A,p.Arg2030Gln | c.1804C>T (p.Arg602Trp) | c.2828G>A (p.Arg943Gln);  c.6089G>A (p.Arg2030Gln) |
| SD10 | c.5318C>T,p.Ala1773Val; c.6308C>A,p.Pro2103His; c.6299G>A,p.Gly2100Glu | c.5318C>T (p.Ala1773Val) | c.6308C>A (p.Pro2103His);  c.6299G>A (p.Gly2100Glu) |
| XT7 | c.3386G>T,p.Arg1129Leu; c.6718A>G,p.Thr2240Ala;  c.4352+61G>A | c.3386G>T (p.Arg1129Leu) | c.6718A>G (p.Thr2240Ala); c.4352+61G>A |
| 5595 | c.3113C>T,p.Ala1038Val; c.5318C>T,p.Ala1773Val; c.5882G>A,p.Gly1961Glu | c.5318C>T (p.Ala1773Val) | c.3113C>T (p.Ala1038Val); c.5882G>A (p.Gly1961Glu) |
| XT10 | c.4926C>G,p.Ser1642Arg;  c.5044_5058del,p.Val1682_1686del;  c.5318C>T,p.Ala1773Val | c.5318C>T (p.Ala1773Val) | c.4926C>G (p.Ser1642Arg); c.5044_5058del,p.Val1682_1686del |
| XT14 | c.3386G>T,p.Arg1129Leu;  c.4253+4C>T;  c.6718A>G,p.Thr2240Ala | c.3386G>T (p.Arg1129Leu) | c.4253+4C>T;  c.6718A>G (p.Thr2240Ala) |
| 5694 | c.3386G>T p.Arg1129Leu;  c.4926C>G p.Ser1642Arg;  c.5044_5058del,p.Val1682_1686del | c.3386G>T (p.Arg1129Leu) | c.4926C>G (p.Ser1642Arg);  c.5044_5058del (p.Val1682_1686del) |
| 5049****** | c.4926C>G,p.Ser1642Arg; c.5044_5058del,p.Val1682_1686delc.4926C>G,p.Ser1642Arg; c.5044_5058del,p.Val1682_1686del | c.4926C>G (p.Ser1642Arg); c.5044_5058del (p.Val1682_1686del) | c.4926C>G (p.Ser1642Arg); c.5044_5058del (p.Val1682_1686del) |

**Supplementary table 3.** Complex *ABCA4* alleles identified in this study. *In three samples (3930, 4214, and 4678), no segregation of variants analysis was performed. **This sample correspond to an individual with uniparental disomy for chromosome 1p. The sample ID correspond to IDs in in supplementary table 1.
