# Supplementary material for: Genotypic spectrum of ABCA4-associated retinal degenerations in 211 unrelated Mexican patients: identification of 22 novel disease-causing variants
Source: Mol Genet Genomics. 2024 Aug 20;299(1):79. doi: 10.1007/s00438-024-02174-x (PMC11335775; doi:10.1007/s00438-024-02174-x)
Supplement: Supplementary file 4 — Supplementary Material 4 [file 438_2024_2174_MOESM4_ESM.docx]

**Supplementary table 4**. ABCA4 pathogenic/likely pathogenic variants identified in a cohort of 211 Mexican individuals with retinal dystrophies.

| **Variant**  **#** | **Localization**  **(E:exón; I:intrón)** | **Nucleotide variant (cDNA)** | **Protein Variant** | **ClinVar (variant ID)** | **LOVD**  **(submission)** |
| --- | --- | --- | --- | --- | --- |
| **1** | E1 | c.52C>T | p.Arg18Trp | **SUB14502950** | 0000453046 |
| **2** | I2 | c.67-1dup |  | SUB14503169 | 0000453047 |
| **3** | E3 | c.179C>T | p.Ala60Val | SUB14503230 | 00451448 |
| **4** | E3 | c.265G>T | p.Glu89* | SUB14503241 | 00451449 |
| **5** | E3 | c.287A>T | p.Asn96Ile | SUB14502950 | 0000453049 |
| **6** | E4 | c.438delT | p.Ile146fs | SUB14503246 | 00451450 |
| **7** | E5 | c.488_491del | p.Leu163Hisfs*18 | SUB14503252 | 00451451 |
| **8** | E6 | c.634C>T | p.Arg212Cys | SUB14503260 | 00451452 |
| **9** | E6 | c.689G>T | p.Cys230Phe | 1804176 | 00408714 |
| **10** | E6 | c.723A>T | p.Glu241Asp | SUB14503291 | 0000123825 |
| **11** | E6 | c.735T>G | p.Tyr245* | SUB14502950 | 00451453 |
| **12** | I6 | c.768+1G>A |  | 1803948 | 00408664 |
| **13** | E7 | Whole deletion |  |  |  |
| **14** | E8 | c.868C>T | p.Arg290Trp | **SUB14503303** | 00077389 |
| **15** | E9 | c.1222C>T | p.Arg408* | **SUB14503309** | 00451454 |
| **16** | E11 | c.1417_1420dup | p.Thr474Asnfs*4 | SUB14503318 | 00240417 |
| **17** | E12 | c.1574T>C | p.Phe525Ser | SUB14503329 | 00451455 |
| **18** | E12 | c.1648G>A | p.Gly550Arg | SUB14503329 | 00451465 |
| **19** | E13 | c.1766G>A | p.Trp589* | SUB14503334 | 00451466 |
| **20** | E13 | c.1798G>T | p.Asp600Tyr | SUB14503345 | 00451467 |
| **21** | E13 | c.1804C>T | p.Arg602Trp | SUB14503359 | 00077392 |
| **22** | E13 | c.1819G>C | p.Gly607Arg | SUB14503402 | 00451468 |
| **23** | E13 | c.1876_1888del | p.Ala626Leufs*19 | 1803949 | 00408666 |
| **24** | I13 | c.1937+1G>A | ---- | SUB14503415 | 00451469 |
| **25** | E14 | c.1994del | p.Tyr665Serfs*5 | SUB14503443 | 00435105 |
| **26** | E14 | c.2023G>A | p.Val675Ile | SUB14503426 | 00451470 |
| **27** | E14 | c.2041C>T | p.Arg681* | SUB14503439 | 00451471 |
| **28** | E15 | c.2267C>T | p.Ser756Phe | SUB14503444 | 00451472 |
| **29** | E15 | c.2297_2299del | p.Gly766del | SUB14503445 | 00451473 |
| **30** | E16 | c.2396C>T | p.Pro799Leu | 858329 | 00408790 |
| **31** | E16 | c.2453G>A | p.Gly818Glu |  | 00077387 |
| **32** | E16 | c.2453G>C | p.Gly818Ala | 1804636 | 00408717 |
| **33** | E16 | c.2522_2530del | p.Gln841_Met843del | 1803950 | 00408699 |
| **34** | E16 | c.2570T>C | p.Leu857Pro | SUB14503448 | 00451474 |
| **35** | E17 | c.2588G>C | p.Gly863Ala | SUB14503451 | 00451475 |
| **36** | E17-23 | deletion | ---- |  |  |
| **37** | E18 | c.2741_2742del | p.His914Argfs*5 | 1383864 | 00408788 |
| **38** | E19 | c.2807del | p.Lys936Argfs*14 | 1429394 | 00408789 |
| **39** | E19 | c.2828G>A | p.Arg943Gln | SUB14503453 | 00077392 |
| **40** | E19 | c.2888delG | p.Gly963Alafs*14 | SUB14503456 | 00451476 |
| **41** | E19 | c.2894A>G | p.Asn965Ser | SUB14503461 | 00077407 |
| **42** | E19 | c.2905A>G | p.Lys969Glu | SUB14503470 | 00451477 |
| **43** | E19 | c.2908del | p.Thr970Profs*7 | 1351564 | 00408791 |
| **44** | E20 | c.3041T>G | p.Leu1014Arg | **SUB14505148** | 00077391 |
| **45** | E21 | c.3056C>T | p.Thr1019Met | SUB14505198 | 00451478 |
| **46** | E21 | c.3113C>T | p.Ala1038Val | SUB14505203 | 00451479 |
| **47** | E22 | c.3210_3211dup | p.Ser1071Cysfs*14 | SUB14505255 | 00451480 |
| **48** | E22 | c.3292C>T | p.Arg1098Cys | SUB14505269 | 00451481 |
| **49** | E22 | c.3308T>G | p.Leu1103Arg | SUB14505272 | 00451482 |
| **50** | E22 | c.3322C>T | p.Arg1108Cys | SUB14505277 | 00451483 |
| **51** | E22 | c.3323G>T | p.Arg1108Leu | SUB14505289 | 00451486 |
| **52** | E23 | c.3352C>T | p.His1118Tyr | SUB14505292 | 00451487 |
| **53** | E23 | c.3383A>G | p.Asp1128Gly | SUB14505302 | 00325457 |
| **54** | E23 | c.3386G>T | p.Arg1129Leu | SUB14505337 | 00077405 |
| **55** | E24 | c.3602T>G | p.Leu1201Arg | SUB14505346 | 00451488 |
| **56** | I25 | c.3608-1G>A | ---- | 1804173 | 00408710 |
| **57** | I26 | c.3814-2A>T | ---- | 1333445 | 00408709 |
| **58** | E27 | c.3898C>T | p.Arg1300* | SUB14505547 |  |
| **59** | E27 | c.3898delC | p.Arg1300fsAspfs*89 | 1803951 | 00408707 |
| **60** | E27 | c.4070C>A | p.Ala1357Glu | SUB14505622 | 00451489 |
| **61** | E28 | c.4139C>T | p.Pro1380Leu | SUB14505647 | 00451490 |
| **62** | E28 | c.4222T>C | p.Trp1408Arg | SUB14505729 | 00451491 |
| **63** | E28 | c.4243dup | p.Thr1415Asnfs*7 | 1804641 | 00408785 |
| **64** | E28 | c.4249_4251del | p.Phe1417del | SUB14505760 | 00077387 |
| **65** | I28 | c.4253+4C>T | ---- | SUB14505769 | 00451492 |
| **66** | E29 | c.4313C>A | p.Pro1438Gln | 1804175 | 00408715 |
| **67** | E29 | c.4328G>A | p.Arg1443His | SUB14505787 | 00077406 |
| **68** | I29 | c.4352+61G>A |  | SUB14505808 | 00436471 |
| **69** | E30 | c.4436G>A | p.Trp1479* | SUB14505828 | 00451493 |
| **70** | E30 | c.4457C>T | p.Pro1486Leu | SUB14505838 | 00451494 |
| **71** | E30 | c.4519G>A | p.Gly1507Arg | SUB14505862 | 00451495 |
| **72** | E30 | c.4537dup | p.Gln1513Profs*42 | SUB14505863 | 00077399 |
| **73** | E31 | c.4558G>C | p.Glu1520Gln | SUB14505867 | 00451496 |
| **74** | E31 | c.4577C>T | p.Thr1526Met | SUB14505869 | 00451497 |
| **75** | E32 | c.4667G>C | p.Arg1556Thr | SUB14505871 | 00077400 |
| **76** | E33 | c.4773G>T | p.Gly1591Gly | SUB14505873 | 00408708 |
| **77** | E34 | c.4793C>A | p.Ala1598Asp | SUB14505876 | 00451498 |
| **78** | E34 | c.4804delA | p.Ile1602Tyrfs*8 | SUB14505882 | 00325457 |
| **79** | I35 | c.4849-1G>A | ---- | SUB14507822 | 00451499 |
| **80** | E35 | c.4852T>C | p.Trp1618Arg | SUB14507833 | 00451500 |
| **81** | E35 | c.4854G>C | p.Trp1618Cys | SUB14507859 | 00325489 |
| **82** | E35 | c.4873C>T | p.His1625Tyr | SUB14507875 | 00451501 |
| **83** | E35 | c.4880del | p.Leu1627Argfs*35 | SUB14507875 | 00451502 |
| **84** | E35 | c.4918C>T | p.Arg1640Trp | SUB14507889 | 00451503 |
| **85** | E35 | c.4919G>A | p.Arg1640Gln | SUB14507913 | 00325485 |
| **86** | E35 | c.4926C>G | p.Ser1642Arg | SUB14507919 | 00436464 |
| **87** | E35 | c.4978C>T | p.Pro1660Ser | SUB14507926 | 00451504 |
| **88** | E36 | c.5044_5058del | p.Val1682_Val1686del | SUB14507936 | 00077406 |
| **89** | E36 | c.5113C>T | p.Arg1705Trp | SUB14507950 | 00451505 |
| **90** | E36 | c.5114G>A | p.Arg1705Gln | SUB14507955 | 00077397 |
| **91** | I36 | c.5196+1G>A | ---- | SUB14507962 | 00325495 |
| **92** | I36 | c.5196+1137G>A | ----- | SUB14507967 | 00451506 |
| **93** | E38 | c.5318C>T | p.Ala1773Val | SUB14507987 | 00077383 |
| **94** | E38 | c.5324T>A | p.Ile1775Asn | SUB14508002 | 00077393 |
| **95** | E38 | c.5333T>A | p.Met1778Lys | SUB14508006 | 00451507 |
| **96** | E38 | c.5335T>C | p.Tyr1779His | SUB14508020 | 00077395 |
| **97** | E38 | c.5413A>G | p.Asn1805Asp | SUB14508022 | 00451508 |
| **98** | I38 | c.5460+3G>A | ---- | 838818 | 00408665 |
| **99** | I38 | c.5461-10T>C | ---- | SUB14508026 | 00451509 |
| **100** | I38 | c.5461-1G>T | ---- | SUB14508031 | 00436473 |
| **101** | E39 | c.5498T>G | p.Leu1833Arg | SUB14508064 | 00451510 |
| **102** | E39 | c.5512C>G | p.His1838Asp | SUB14508080 | 00451511 |
| **103** | E39 | c.5527C>T | p.Arg1843Trp | SUB14508084 | 00451512 |
| **104** | I40 | c.5714+5G>A | ----- | SUB14508101 | 00451513 |
| **105** | E41 | c.5819T>C | p.Leu1940Pro | SUB14508118 | 00325475 |
| **106** | E41 | c.5824G>C | p.Glu1942Gln | SUB14508130 | 00451514 |
| **107** | E42 | c.5843C>T | p.Pro1948Leu | SUB14508170 | 00451515 |
| **108** | E42 | c.5882G>A | p.Gly1961Glu | SUB14508176 | 00451516 |
| **109** | E43 | c.5951T>G | p.Met1984Arg | SUB14508186 | 00451517 |
| **110** | E44 | c.6089G>A | p.Arg2030Gln | SUB14508191 | 00451518 |
| **111** | E44 | c.6094C>T | p.His2032Tyr | SUB14508195 | 00451519 |
| **112** | E44 | c.6119G>A | p.Arg2040Gln | SUB14508200 | 00451520 |
| **113** | E45 | c.6220G>T | p.Gly2074Cys | SUB14508205 | 00451521 |
| **114** | E45 | c.6221G>T | p.Gly2074Val | SUB14508212 | 00077401 |
| **115** | E45 | c.6148G>C | p.Val2050Leu | SUB14508220 | 00325470 |
| **116** | I45 | c.6282+3A>T | ---- | SUB14508232 | 00240419 |
| **117** | E46 | c.6299G>A | p.Gly2100Glu | SUB14508247 | 00408784 |
| **118** | E46 | c.6306C>A | p.Asp2102Glu | SUB14508272 | 00451522 |
| **119** | E46 | c.6308C>A | p.Pro2103His | 1172702 | 00408783 |
| **120** | E46 | c.6320G>A | p.Arg2107His | SUB14508281 | 00451523 |
| **121** | E46 | c.6339C>G | p.Ile2113Met | SUB14508298 | 00451524 |
| **122** | E46 | c.6383A>G | p.His2128Arg | SUB14508317 | 00077402 |
| **123** | E47 | c.6394G>T | p.Glu2132* | SUB14508338 | 00451525 |
| **124** | E47 | c.6397T>C | p.Cys2133Arg | SUB14508348 | 00451526 |
| **125** | E47 | c.6401A>G | p.Glu2134Gly | 1510982 | 00408716 |
| **126** | E47 | c.6446G>C | p.Arg2149Pro | 1480664 | 00408669 |
| **127** | E48 | c.6686T>C | p.Leu2229Pro | SUB14508360 | 00451527 |
| **128** | E48 | c.6718A>G | p.Thr2240Ala | SUB14508373 | 00451528 |
